# The effect of preoperative sodium-glucose cotransporter 2 inhibitors on the incidence of perioperative metabolic acidosis: A retrospective cohort study

**DOI:** 10.1186/s12902-022-01126-z

**Published:** 2022-08-20

**Authors:** Yudai Iwasaki, Yusuke Sasabuchi, Sho Horikita, Taku Furukawa, Junji Shiotsuka, Alan Kawarai Lefor, Masamitsu Sanui

**Affiliations:** 1 Department of Anesthesiology and Critical Care, Saitama Medical Center, Jichi Medical University, 1-847 Amanuma-cho, Omiya-ku, Saitama City, Saitama 330-8503 Japan; 2 Department of Anesthesiology and Perioperative Medicine, Tohoku University Graduate School of Medicine, 1-1 Seiryo-machi, Aoba-ku, Sendai, Miyagi 980-8574 Japan; 3 Data Science Center, Jichi Medical University, 3311-1, Yakushiji, Shimotsuke, Tochigi 329-0498 Japan; 4 Department of Surgery, Jichi Medical University, 3311-1, Yakushiji, Shimotsuke, , Tochigi 329-0498 Japan

**Keywords:** Sodium-glucose cotransporter 2 inhibitors, Euglycemic diabetic ketoacidosis, Perioperative metabolic acidosis, Diabetes mellitus, Intensive care unit

## Abstract

**Background:**

Sodium-glucose cotransporter 2 inhibitors are a novel class of anti-hyperglycemic agents. Although several cases of perioperative euglycemic diabetic ketoacidosis have been linked to these medications, the association remains unclear. This study aimed to examine the association between sodium-glucose cotransporter 2 inhibitor use and the incidence of perioperative metabolic acidosis with euglycemia, the surrogating outcome of perioperative euglycemic diabetic ketoacidosis.

**Method:**

This was a retrospective, matched cohort study, which was conducted in the intensive care unit of a tertiary care facility in Japan. We identified patients aged 20 years or older with diabetes mellitus who received pharmacologic therapy and were admitted to the intensive care unit after elective surgery between April 2014 and March 2019. We extracted the following data from the electronic medical record for matching: age, sex, surgery year, surgical site, hemoglobin A1c level, and prescription for sodium-glucose cotransporter 2 inhibitors.

Eligible patients were divided into two groups, those who were prescribed sodium-glucose cotransporter 2 inhibitors (SGLT2-i group) and those who were not (control group). For each patient in the SGLT2-i group, we randomly selected four patients from the control group matched for the extracted characteristics. The primary outcome was the incidence of metabolic acidosis with an elevated anion gap and euglycemia. The secondary outcome was the lowest pH value of each patient during their ICU stay.

**Results:**

A total of 155 patients were included in this study. Patients receiving sodium-glucose cotransporter 2 inhibitors had comparable characteristics to control participants; however, the proportions of patients undergoing dialysis were not similar. Metabolic acidosis with euglycemia was seen in 7/31 (22.6%) patients receiving sodium-glucose cotransporter 2 inhibitors and in 10/124 (8.1%) control patients (*p* = 0.047).

**Conclusions:**

This study shows that the use of sodium-glucose cotransporter 2 inhibitors is associated with a significantly higher incidence of metabolic acidosis with euglycemia. Patients receiving sodium-glucose cotransporter 2 inhibitors who are scheduled to undergo invasive surgical procedures should be closely monitored for the development of euglycemic diabetic ketoacidosis.

**Supplementary Information:**

The online version contains supplementary material available at 10.1186/s12902-022-01126-z.

## Background

Diabetes mellitus is present in approximately 10–15% of patients who undergo surgical procedures and is a global public health challenge [1, 2]. Several pharmacologic agents have recently been developed to control diabetes mellitus. Sodium-glucose cotransporter 2 (SGLT2) inhibitors are a novel class of anti-hyperglycemic agents. SGLT is present in the proximal tubule of the kidney and functions as a cotransporter of sodium and glucose from the blood to the urine. The hypoglycemic effect of SGLT2 inhibitors is exerted by inhibiting SGLT [3]. SGLT2 inhibitors are recommended as second-line drugs in the Canadian guidelines for the treatment of patients with diabetes mellitus [4]. Currently, the use of SGLT2 inhibitors is increasing worldwide, including in Japan.

Although SGLT2 inhibitors have several organ-protective effects, [5, 6] they are associated with an increased incidence of side effects, including dehydration, dry mouth, urinary tract infections, and euglycemic diabetic ketoacidosis (eDKA), the most severe adverse effect of the drug. eDKA has been reported to occur in patients taking SGLT2 inhibitors by increasing glucagon, which promotes ketogenesis and lipolysis in the liver, ultimately resulting in glucose release into the urine [7].

eDKA has been reported in approximately 0.1% of patients who take SGLT2 inhibitors [8] and eDKA is considered to be a rare complication. Nonetheless, there are several case reports of perioperative eDKA that is likely associated with SGLT2 inhibitor use. A systematic review of perioperative diabetic ketoacidosis reported 42 patients with eDKA in the perioperative period [9]. However, this systematic review included only case reports or case series and did not provide epidemiological evidence of an association between SGLT2 inhibitor use and perioperative eDKA. Furthermore, ketoacidosis has been reported in 3% of critically ill patients with diabetes mellitus, [10] suggesting that it may occur due to diabetes mellitus itself rather than from the medication prescribed. It remains unclear whether the use of SGLT2 inhibitors is associated with perioperative ketoacidosis.

Therefore, a retrospective observational study was conducted for patients with diabetes who underwent elective surgery. Originally, we aimed to evaluate whether SGLT2 inhibitor use is associated with the incidence of perioperative diabetic ketoacidosis. However, blood ketone and urine ketone body levels are not routinely measured at our institute. Therefore, in this study, we aimed to examine the association between SGLT2 inhibitor use and the incidence of perioperative metabolic acidosis with a high anion gap and euglycemia, the surrogate outcome of perioperative euglycemic diabetic ketoacidosis. We hypothesized that the use of SGLT2 inhibitors is associated with an increased incidence of metabolic acidosis.

## Methods

### Study design and patient population

This retrospective matched-pair cohort study was conducted in the intensive care unit (ICU) of Saitama Medical Center of Jichi Medical University, a tertiary care facility in Japan. Approximately 2000 patients are admitted annually to this ICU. This study was approved by the Jichi Medical University Bioethics Committee the review board of this hospital on March 24, 2020 (The board name: Jichi Medical University Bioethics Committee for Clinical Research, Saitama Medical Center, Approval number: S19-156). This study adheres to the principles of the Declaration of Helsinki. The requirement for written consent was waived because of the retrospective study design.

### Patient selection

Patients aged 20 years or older with type 1 or type 2 diabetes mellitus who were admitted to the ICU after elective surgery between April 2014 and March 2019 were identified. Patients who underwent emergency surgery or surgery with cardiopulmonary bypass were excluded. Subsequently, patients receiving pharmacological therapy for diabetes mellitus were selected. Pharmacological therapy status was confirmed if at least one antidiabetic drug was prescribed. Data regarding the following characteristics were extracted from the electronic medical record: age, sex, surgery year, surgical type (cardiovascular, respiratory, urological, neurosurgery, abdominal, or orthopedic surgery), hemoglobin A1c (HbA1c) level, and SGLT2 inhibitor prescription.

### One to four pair matching of patients using SGLT2 inhibitors and patients not using SGLT2 inhibitors

Eligible patients were divided into two groups: those taking SGLT2 inhibitors (SGLT2-i group) preoperatively and those who did not (control group). For each patient in the SGLT2-i group, four patients of the same sex, age category (age ≤ 49, 50–59, 60–69, 70–79, and ≥ 80 years old), year of surgery (2014, 2015, 2016, 2017, 2018, and 2019), surgery type, and HbA1c category (HbA1c < 6%, ≥ 6% and < 7%, ≥ 7% and < 8%, ≥ 8% and < 9%, and ≥ 9%) were randomly selected from the control group and matched. In this hospital, the last instance of prescription of SGLT2 inhibitors was the day before surgery during the study period. This medication was discontinued (withdrawal period) in 24 h. Resumption of the drug was left to the discretion of the attending physician.

### Data collection

The following characteristics were reviewed for matched patients: sex; weight; height; body mass index; Acute Physiology and Chronic Health Evaluation II (APACHE II) scores; [11] surgery year; surgical type; type of diabetes; HbA1c level; type of SGLT2 inhibitors; use of other antidiabetic drugs; serum creatinine; estimated glomerular filtration rate (eGFR) calculated by the GFR equation for the Japanese population; [12] and history of cancer, hypertension, heart failure, myocardial infarction, stroke, peripheral arterial disease, and maintenance dialysis. In this ICU, arterial blood gas analysis was performed 3–6 times daily for each patient. All arterial blood gas analysis data collected during the patients’ ICU stay was included. We could not collect data on blood gas analysis after ICU discharge because almost all patients did not have their blood gas levels analyzed in the medical ward.

### Definition of main outcomes

The primary outcome was the incidence of metabolic acidosis with an elevated anion gap and euglycemia during the ICU stay. The primary aim was to evaluate the incidence of eDKA. However, since blood ketone and urine ketone body levels were not measured, the outcome of interest was the incidence of metabolic acidosis with a high anion gap and euglycemia instead of the incidence of eDKA. The definition of this outcome was pH < 7.3, anion gap of > 12 mmol/L, PaCO_2_ < 45 mmHg, and glucose levels of < 252 mg/dL, based on previously reported values [13]. We did not include lactate levels of > 5 mmol/L as the primary outcome, as these values suggest lactic acidosis, not eDKA [14]. The secondary outcome was the proportion of metabolic acidosis, ICU stay, the lowest pH value for each patient during the ICU stay, and other parameters of the blood gas analysis which showed the lowest pH. Metabolic acidosis was defined in accordance with the primary outcome, notwithstanding the blood glucose level, which corresponded with moderate or severe metabolic acidosis [15]. Suitability of this model was assessed by a diagnostic plot and the locally weighted scatterplot smoother (LOWESS) by using the dependent value and each independent value.

### Statistical analysis

Normally distributed continuous variables are reported as the mean and standard deviation, whereas non-normally distributed continuous variables are reported as the median with the first and third quartiles. Categorical variables are reported as counts and percentages. After one-to-four matching, characteristics were compared between the groups. Normally and non-normally distributed continuous variables were compared using the *t*-test and Man–Whitney U test, respectively. Categorical variables were compared using Fisher’s exact test. Two-sided p-values of < 0.05 were considered statistically significant.

Multivariable linear regression analysis was conducted to evaluate whether SGLT2 inhibitor use was associated with the lowest pH during the ICU stay. The following factors are included in the model: binary variables of SGLT2 inhibitor use, insulin use, metformin use, and maintenance dialysis; categorical variable of surgery type; and continuous variables of eGFR, age, duration of surgery, APACHE2 score, and HbA1c levels. These factors were selected according to their clinical impact on outcomes.

Subgroup analysis was conducted by excluding patients with end-stage kidney disease, which was defined as an eGFR of ≤ 30 ml/min/1.73m^2^. Multiple imputation was scheduled to be conducted if the proportion of missing data was ≥ 5% [16]. If the proportion of missing data was < 5%, the listwise deletion method was used. All analyses were conducted using R version 4.1.1 software (2021–08-10).

## Results

### Study population

**Fig. 1:**
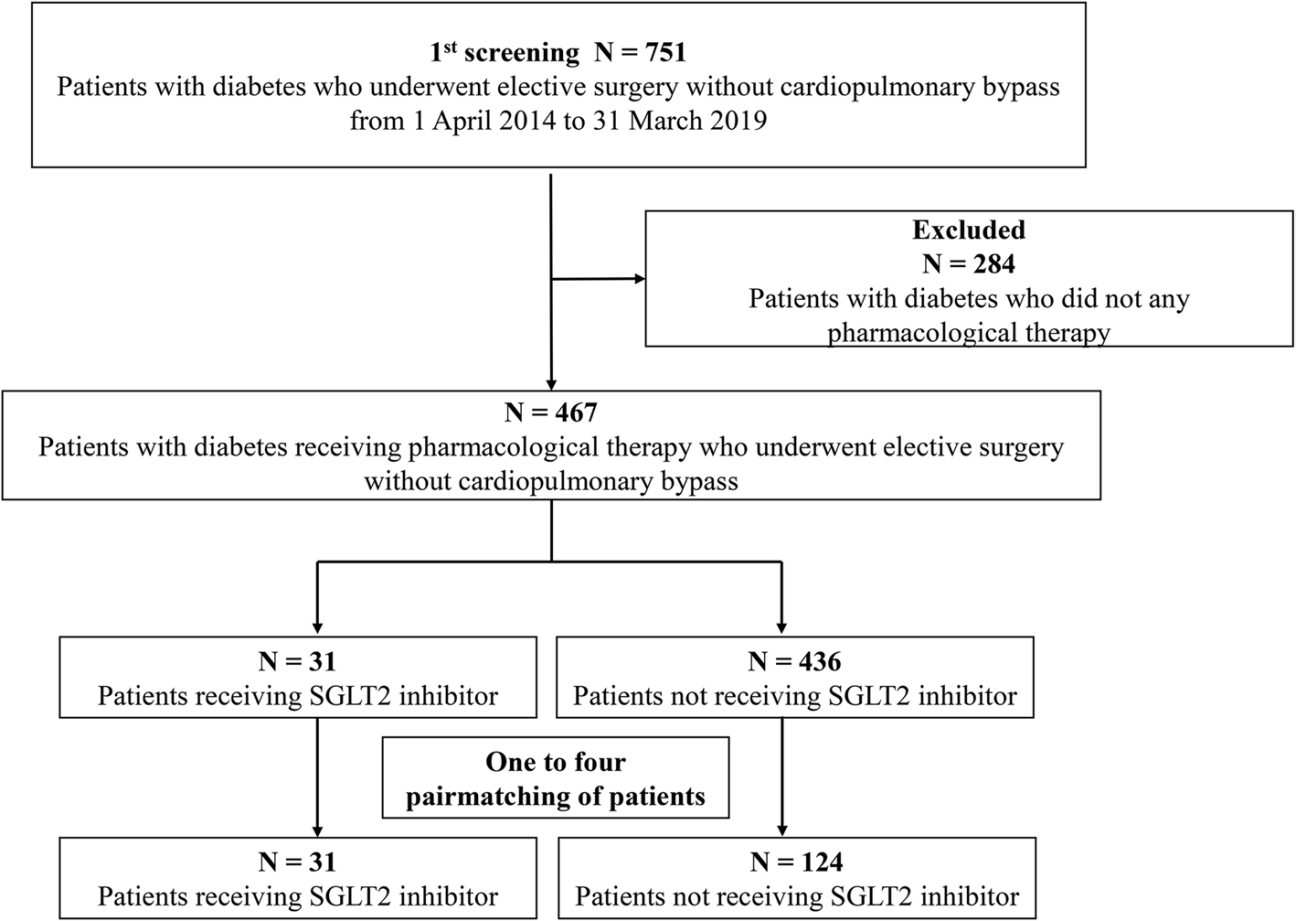
Flowchart of screening and matched-pair selection of patients with diabetes who underwent elective surgery. SGLT2 inhibitor = sodium-glucose cotransporter-2 inhibitor

A total of 751 patients were screened for this study. Two hundred and eighty-four patients were excluded because they did not receive pharmacological therapy for diabetes mellitus, and the remaining 467 patients were eligible for this study. After matching, 155 patients were included (Fig. 1). The patient characteristics are presented in Supplemental Digital Content 1. All patients in the study and control groups had type 2 diabetes, except three and one patient in the control group, who had type 1 and pancreatic diabetes, respectively. The proportions of patients that underwent maintenance dialysis or presented preoperatively with myocardial infarction were significantly different between the two groups, and there were no significant between-group differences in other parameters. Empagliflozin was used for 24 patients in the SGLT2-i group and was the most frequently used SGLT inhibitor. All patients stopped taking the SGLT2 inhibitor on the day of surgery.

### Estimation of main outcomes

The parameters of interest are presented in Table 1. A total of 1527 blood gas analyses were conducted in this cohort. Twenty blood gas analyses showed evidence of lactic acidosis (SGLT2-i group: 2 patients and 7 readings; control group: 6 patients and 13 readings) and were excluded from analysis for the primary outcome. Analyses of blood gas data for the lowest pH value in each patient revealed missing lactate level data in four (2.5%) patients. Therefore, we performed listwise method analysis. All other data were complete. Metabolic acidosis with euglycemia occurred in 7/31 and 10/124 (22.6% vs. 8.1%, *p* = 0.047) patients in the SGLT2-i and control groups, respectively. The proportion of patients with metabolic acidosis was not different between the two groups. ICU length of stay was comparable in both groups. Bicarbonate levels, base excess and anion gap values were significantly different between the two groups.

**Table 1:** Comparison of perioperative outcomes

Outcomes	SGLT2-i (*n* = 31)	Matched Controls (*n* = 124)	*p-*value
Metabolic acidosis with euglycemia, n, (%)	7 (22.6)	10 (8.1)	**0.047**
Metabolic acidosis, n (%)	7 (22.6)	11 (8.9)	0.054
ICU stay, hours, median [IQR]	43.00 [21.00, 47.50]	22.00 [18.00, 49.00]	0.061
Blood gas analysis of the lowest pH at ICU admission			
Lowest pH, median [IQR]	7.33 [7.30, 7.36]	7.35 [7.31, 7.38]	0.12
PaCO_2_, mmHg, median [IQR]	41.00 [36.00, 44.65]	43.45 [39.88, 47.02]	**0.013**
Glucose, mg/dL, median [IQR]	147.00 [123.00, 174.50]	161.50 [124.75, 201.25]	0.19
Lactate, mmol/L, median [IQR]	1.39 [1.15, 1.81]	1.30 [1.08, 1.80]	0.71
Sodium, mEq/L, median [IQR]	137.00 [134.80, 138.10]	135.95 [134.17, 137.60]	0.22
Potassium, mEq/L, median [IQR]	4.28 [3.88, 4.54]	4.08 [3.86, 4.30]	0.15
Chloride, mEq/L, mean (SD)	106.19 (4.06)	104.94 (3.23)	0.068
Bicarbonate, mEq/L, median [IQR]	22.00 [18.55, 23.20]	23.35 [21.70, 24.90]	** < 0.001**
Base excess, mmol/L. median [IQR]	-3.70 [-8.00, -2.05]	-2.55 [-3.90, -0.85]	**0.002**
Anion gap, mEq/L, median [IQR]	14.20 [11.45, 16.75]	11.65 [9.60, 13.50]	**0.004**

*Abbreviation*: *ICU* Intensive care unit, *IQR* Interquartile range, *PaCO*_*2*_ Partial pressure of arterial carbon dioxide, *SD* Standard deviation. Significant *p*-values (*p* < 0.05) are given in bold

### Subgroup analysis

Supplemental Digital Content 2 and Table 2 present the results of the subgroup analyses. The subgroups, excluding patients with end-stage kidney disease, included 27/31 and 95/124 patients in the SGLT2-i and control groups, respectively. There were three (2.4%) cases of missing lactate level data in these subgroups and the listwise deletion method was used. Patient baseline characteristics were comparable in both groups, except for the frequency of preoperative heart failure. The difference in the incidence of acidosis between the two groups was more pronounced in the subgroup analysis than in the main analysis.

**Table 2:** Comparison of perioperative outcomes (excluding patients with severe chronic kidney disease)

Outcomes	SGLT2-i (*n* = 27)	Matched Controls (*n* = 95)	*p-*value
Metabolic acidosis with euglycemia, n (%)	6 (22.2)	6 (6.3)	**0.024**
Metabolic acidosis, n (%)	6 (22.2)	7 (7.4)	**0.038**
ICU stay, hours, median [IQR]	23.00 [21.00, 47.50]	22.00 [18.00, 54.50]	0.15
Blood gas analysis of the lowest pH at ICU admission			
Lowest pH, median [IQR]	7.33 [7.30, 7.36]	7.35 [7.32, 7.38]	0.11
PaCO_2_, mmHg, median [IQR]	40.20 [36.00, 44.35]	44.00 [40.05, 46.90]	**0.002**
Glucose, mg/dL, median [IQR]	144.00 [119.50, 171.00]	164.00 [133.50, 203.00]	**0.027**
Lactate, mmol/L, median [IQR]	1.41 [1.10, 1.81]	1.31 [1.08, 1.86]	0.94
Sodium, mEq/L, median [IQR]	137.20 [135.10, 138.35]	136.20 [134.45, 137.90]	0.20
Potassium, mEq/L, median [IQR]	4.14 [3.76, 4.52]	4.05 [3.88, 4.24]	0.31
Chloride, mEq/L, mean (SD)	106.59 (4.02)	105.23 (3.29)	0.074
Bicarbonate, mEq/L, median [IQR]	22.00 [18.55, 23.20]	23.70 [22.60, 25.10]	** < 0.001**
Base excess, mmol/L. median [IQR]	-3.70 [-6.90, -2.05]	-2.20 [-3.45, -0.55]	** < 0.001**
Anion gap, mEq/L, median [IQR]	13.30 [11.45, 16.55]	10.70 [9.10, 12.50]	**0.002**

*Abbreviation*: *ICU* Intensive care unit, *IQR* Interquartile range, *PaCO*_*2*_ Partial pressure of arterial carbon dioxide, *SD* Standard deviation. Significant *p*-values (*p* < 0.05) are given in bold

### Association of SLGT-2i with main outcomes

Supplemental Digital Content 3 shows the results of multivariable linear regression analysis of factors associated with the lowest pH during the ICU stay. In addition to P_a_CO_2_ and anion gap values, SGLT2 inhibitor use, duration of surgery, and APACHE2 score were independently associated with the lowest pH during the ICU stay. Among drugs included in the model, only SGLT2 inhibitors were associated with pH in this analysis (coefficient: -0.026, 95% confidence interval: -0.041 to -0.01, *p* < 0.01). A diagnostic plot showed normal distribution of residual error. LOWESS of the lowest pH and independent variables, which was statistically associated with the pH, showed an almost linear regression.

## Discussion

In this study, we investigated the incidence of postoperative metabolic acidosis after elective surgery in patients with diabetes mellitus; specifically, we compared the incidence in patients treated with SGLT2 inhibitors compared to those who were not. We found that the preoperative use of SGLT2 inhibitors was associated with an increased incidence of metabolic acidosis with euglycemia during the ICU stay after elective surgery. The proportion of patients with metabolic acidosis did not differ between the two groups, and metabolic acidosis with hyperglycemia was present in only one patient of the control group. The results of the multivariable linear regression analysis suggest that SGLT2 inhibitors are the only medication that affected pH among the medications included in the model. Although we could only detect the incidence of metabolic acidosis with euglycemia, these results suggest that perioperative SGLT2 inhibitor use is associated with eDKA—one of the major causes of mortality among patients with diabetes [17].

To the best of our knowledge, this is the first study to evaluate the relationship between the preoperative use of SGLT2 inhibitors and the incidence of perioperative metabolic acidosis with euglycemia. A previous systematic review suggested that perioperative eDKA is a common condition in patients treated with SGLT2 inhibitors [9]. However, since that systematic review only included case reports or case series, the relationship between eDKA and SGLT2 inhibitors remained unclear. In the present study, we performed an adjustment for confounding factors using one-to-four matching for patients treated with an SGLT2 inhibitor and controls. We found an association between SGLT2 inhibitor use and an increased incidence of postoperative metabolic acidosis with euglycemia. A previous study showed that patients are exposed to food deprivation and dehydration during the perioperative period, and thus they may be vulnerable to developing eDKA [18].

In this study, both groups had a high incidence of metabolic acidosis with euglycemia compared to that reported in a previous study [10]. There are two reasons which may account for the high incidence of metabolic acidosis in this study. First, the hospital is among the leading cardiovascular centers in Japan, and approximately half of the patients in this study underwent cardiovascular surgery, which is an invasive surgical procedure. Stress induced by the surgical procedure may increase the incidence of metabolic acidosis [19]. Second, in this study, patients stopped receiving SGLT2 inhibitors on the day of surgery to decrease the risk of perioperative eDKA [18]. The Japanese package inserts distributed with prescription drugs recommend that these drugs be stopped on the day of surgery. The patients in this study followed these recommendations. In contrast, the United States Food and Drug Administration (USFDA) recommends that this drug be stopped 3 or 4 days before surgery [20]. This withdrawal period of SGLT2 inhibitors was determined according to their half-life, which is approximately 12 h [21–24]. Based on this half-life, patients needed 3 or 4 days after withdrawal of the drug for it to disappear from the body. The patients in this study had a withdrawal period that was shorter than that of patients following the USFDA recommendation. The short withdrawal period may have increased the incidence of metabolic acidosis identified in this study because the short withdrawal duration of only 24 h suggests the possibility of drug residues in the body.

These findings suggest that meticulous perioperative monitoring of arterial blood gas results may be needed in patients with diabetes mellitus who are treated with SGLT2 inhibitors. Patients who undergo highly invasive procedures (e.g., cardiovascular surgery) may be at a higher risk of developing eDKA. Withdrawal of medication on the day of surgery may be insufficient to prevent eDKA because of the half-life of SGLT2 inhibitors. When these medications are withdrawn prior to the day before surgery, monitoring of preoperative glucose levels and use of alternative drugs to control blood glucose might be needed for better preoperative management.

This study has several limitations. First, the small sample size and the retrospective and observational nature of the study design remain potential biases and may have caused restrictions in performing analyses. Our original database, which was used for pair matching, did not include the information to adjust the confounding factors, which may have introduced some selection biases into this study. SGLT2 inhibitor use may reduce the incidence of cardiovascular events [5, 25, 26] and cause a decline in renal function [6, 26–28]. Moreover, SGLT2 inhibitors may not be used to treat patients with anuria because the drug affects the proximal tubules of the kidneys and promotes glucose excretion. These characteristics might distort the patients’ background of the two groups. However, a subgroup analysis of patients without chronic kidney disease showed larger between-group differences for the primary outcome, suggesting that the overall findings are robust. We could not conduct several analyses, including binomial logistic regression, propensity score matching, and interaction analysis, to examine the perioperative effects of SGLT2 inhibitors because of the small number of participants in this study.

Second, our medical records did not include the duration of diabetes, level of ketone bodies, and data on blood gas analysis after ICU discharge. As regards ketone bodies, the ICU did not conduct routine urinalyses, and measurement of ketone bodies in the blood is difficult in Japanese hospitals because the required devices are not paid for by the national insurance system. Therefore, we assessed the incidence of metabolic acidosis without accounting for hyperglycemia. However, ketoacidosis is among the common causes of a high anion gap in patients with metabolic acidosis [29]. These results provide preliminary evidence of an association between SGLT2 inhibitors and the incidence of perioperative eDKA. Lack of the blood gas analysis data after ICU discharge made it difficult to estimate the incidence rate of the primary outcome. However, this study design was not based on long-term cohort follow-up. To estimate the incidence rate, prospective studies are needed to evaluate time to event of eDKA.

Third, the external validity of this study remains unclear. In December 2020, the Japan Diabetes Society recommended a preoperative withdrawal period of SGLT2 inhibitors to match that of the USFDA guideline [30]. This period was set according to the half-life of this medication [21–24]. However, in this study, the patients did not stop taking SGLT2 inhibitors 3 or 4 days before surgery. This delay in withdrawal might have affected the incidence of metabolic acidosis. Further studies are required to estimate the incidence of ketoacidosis in contexts where an appropriate withdrawal period is observed. Large clinical trials are needed for meticulous evaluation to overcome these limitations. In Japan, a multicenter observational study examining the incidence of postoperative ketoacidosis associated with SGLT2 inhibitor use is on-going [31], which might clarify the unresolved issues.

## Conclusions

This study shows that the use of SGLT2 inhibitors is associated with an increased incidence of metabolic acidosis with euglycemia. Patients receiving SGLT2 inhibitors who are scheduled to undergo invasive surgical procedures should be closely monitored by blood gas analysis for the development of eDKA, specifically, in patients with a short interval before surgery after withdrawal of these medications.

## Supplementary Information

**Additional file 1:** **Supplemental Digital Content 1.** Baseline demographic and clinical characteristics of all patients.

**Additional file 2: Supplemental Digital Content 2.** Baseline demographic and clinical characteristics (excluding patients with severe chronic kidney disease).

**Additional file 3:** **Supplemental Digital Content 3.** Multivariable linear regression analysis to identify factors associated with the lowest pH during intensive care unit stay.

## Data Availability

The datasets used and analysed during the current study are available from the corresponding author on reasonable request.

## Abbreviations

APACHE II: Acute physiology and chronic health evaluation II
eDKA: Euglycemic diabetic ketoacidosis
eGFR: Estimated glomerular filtration rate
HbA1c: Hemoglobin a1c
ICU: Intensive care unit
SGLT2: Sodium-glucose cotransporter 2
