# Supplementary material for: The effect of preoperative sodium-glucose cotransporter 2 inhibitors on the incidence of perioperative metabolic acidosis: A retrospective cohort study
Source: BMC Endocr Disord. 2022 Aug 20;22:209. doi: 10.1186/s12902-022-01126-z (PMC9392326; doi:10.1186/s12902-022-01126-z)
Supplement: Supplementary file 1 — Additional file 1: Supplemental Digital Content 1. Baseline demographic and clinical characteristics of all patients. [file 12902_2022_1126_MOESM1_ESM.docx]

Supplemental Digital Content 1. Baseline demographic and clinical characteristics of all patients

| Characteristics | SGLT2-i (n=31) | Matched controls (n=124) | *p-*value |
| --- | --- | --- | --- |
| Type of diabetes, n (%) |  |  | 1.0 |
| Type 1 diabetes | 0 (0.0) | 3 (2.4) |  |
| Type 2 diabetes | 31 (100.0) | 120 (96.8) |  |
| Pancreatic diabetes | 0 (0.0) | 1 (0.8) |  |
| Year of surgery, n (%) |  |  | 0.99 |
| 2014 | 1 (3.2) | 7 (5.6) |  |
| 2015 | 1 (3.2) | 5 (4.0) |  |
| 2016 | 2 (6.5) | 9 (7.3) |  |
| 2017 | 9 (29.0) | 38 (30.6) |  |
| 2018 | 15 (48.4) | 51 (41.1) |  |
| 2019 | 3 (9.7) | 14 (11.3) |  |
| Age, years, median [IQR] | 69.00 [62.50, 74.00] | 71.00 [66.00, 76.00] | 0.17 |
| HbA1c, %, mean (SD) | 7.75 (1.38) | 7.55 (1.25) | 0.44 |
| Creatinine, mg/dL, median [IQR] | 0.87 [0.75, 1.04] | 0.92 [0.73, 1.48] | 0.29 |
| eGFR, ml/min/1.73m^2^, median [IQR] | 65.77 [53.65, 76.97] | 60.14 [33.53, 76.03] | 0.17 |
| Male, n (%) | 23 (74.2) | 89 (71.8) | 1 |
| Type of Surgery, n (%) |  |  | 0.94 |
| Abdominal | 5 (16.1) | 17 (13.7) |  |
| Thoracic | 7 (22.6) | 33 (26.6) |  |
| Cardiovascular | 15 (48.4) | 61 (49.2) |  |
| Orthopedics | 1 (3.2) | 3 (2.4) |  |
| Urology | 3 (9.7) | 10 (8.1) |  |
| Body weight, kg, median [IQR] | 63.20 [55.00, 73.00] | 60.85 [53.70, 68.75] | 0.26 |
| Height, cm, mean (SD) | 164.31 (10.02) | 161.45 (8.44) | 0.11 |
| Body mass index, kg/m^2^, median [IQR] | 23.60 [20.64, 26.08] | 23.35 [20.66, 25.87] | 0.72 |
| Duration of surgery, minutes,  median [IQR] | 265.00 [173.00, 342.50] | 269.50 [176.75, 371.75] | 0.74 |
| APACHE2 score (median [IQR]) | 12.00 [9.50, 13.50] | 13.00 [10.00, 16.00] | 0.11 |
| Antidiabetic Medication |  |  |  |
| SGLT2 inhibitor, n (%) |  |  |  |
| Ipragliflozin | 2 (6.5) | NA |  |
| Empagliflozin | 24 (77.4) | NA |  |
| Canagliflozin | 2 (6.5) | NA |  |
| Dapagliflozin | 1 (3.2) | NA |  |
| Luseogliflozin | 2 (6.5) | NA |  |
| Insulin, n (%) | 18 (58.1) | 60 (48.4) | 0.42 |
| Metformin, n (%) | 7 (22.6) | 15 (12.1) | 0.15 |
| Sulfonylurea, n (%) | 8 (25.8) | 22 (17.7) | 0.32 |
| Dipeptidyl peptidase-4 inhibitor, n (%) | 20 (64.5) | 80 (64.5) | 1 |
| Thiazolidine, n (%) | 2 (6.5) | 2 (1.6) | 0.18 |
| GLP1 receptor agonist, n (%) | 2 (6.5) | 8 (6.5) | 1 |
| Alpha-glucosidase inhibitor, n (%) | 5 (16.1) | 11 (8.9) | 0.32 |
| Glinide, n (%) | 3 (9.7) | 5 (4.0) | 0.20 |
| Previous-onset comorbidities |  |  |  |
| Cancer, n (%) | 16 (51.6) | 63 (50.8) | 1 |
| Hypertension, n (%) | 21 (67.7) | 62 (50.0) | 0.11 |
| Heart failure, n (%) | 10 (32.3) | 23 (18.5) | 0.14 |
| Myocardial infarction, n (%) | 19 (61.3) | 49 (39.5) | **0.04** |
| Stroke, n (%) | 4 (12.9) | 12 (9.7) | 0.53 |
| Peripheral arterial disease, n (%) | 7 (22.6) | 23 (18.5) | 0.62 |
| Other kidney disease, n (%) | 3 (9.7) | 10 (8.1) | 0.72 |
| Maintenance dialysis, n (%) | 0 (0.0) | 21 (16.9) | **0.01** |

Abbreviations: HbA1c = hemoglobin A1c, eGFR = estimated glomerular filtration rate, APACHE2 = Acute Physiology and Chronic Health Evaluation II, SGLT2 = Sodium-Glucose Cotransporter-2, GLP1 = glucagon-like peptide-1

Significant *p*-values (*p* < 0.05) are given in bold.
