# Supplementary material for: The effect of preoperative sodium-glucose cotransporter 2 inhibitors on the incidence of perioperative metabolic acidosis: A retrospective cohort study
Source: BMC Endocr Disord. 2022 Aug 20;22:209. doi: 10.1186/s12902-022-01126-z (PMC9392326; doi:10.1186/s12902-022-01126-z)
Supplement: Supplementary file 2 — Additional file 2: Supplemental Digital Content 2. Baseline demographic and clinical characteristics (excluding patients with severe chronic kidney disease). [file 12902_2022_1126_MOESM2_ESM.docx]

Supplemental Digital Content 2. Baseline demographic and clinical characteristics (excluding patients with severe chronic kidney disease)

| Charanteristics | SGLT2-i (n=27) | Matched Controls (n=95) | *p-*value |
| --- | --- | --- | --- |
| Type of diabetes, n (%) |  |  |  |
| Type 1 diabetes | 0 (0.0) | 1 (1.1) | 1.00 |
| Type 2 diabetes | 27 (100.0) | 93 (97.9) |  |
| Pancreatic diabetes | 0 (0.0) | 1 (1.1) |  |
| Year of surgery, n (%) |  |  | 0.88 |
| 2014 | 1 (3.7) | 5 (5.3) |  |
| 2015 | 1 (3.7) | 5 (5.3) |  |
| 2016 | 0 (0.0) | 5 (5.3) |  |
| 2017 | 7 (25.9) | 29 (30.5) |  |
| 2018 | 15 (55.6) | 40 (42.1) |  |
| 2019 | 3 (11.1) | 11 (11.6) |  |
| Age, years, median [IQR] | 69.00 [64.50, 74.00] | 71.00 [67.50, 77.00] | 0.14 |
| HbA1c, %, mean (SD) | 7.76 (1.44) | 7.61 (1.21) | 0.59 |
| Creatinine, mg/dL, median [IQR] | 0.86 [0.75, 0.95] | 0.80 [0.70, 0.95] | 0.97 |
| eGFR, ml/min/1.73m^2^, median [IQR] | 68.18 [59.38, 78.56] | 65.20 [54.27, 80.08] | 0.69 |
| Male, n (%) | 21 (77.8) | 71 (74.7) | 1 |
| Surgical Type, n (%) |  |  | 0.65 |
| Abdominal | 3 (11.1) | 14 (14.7) |  |
| Thoracic | 7 (25.9) | 32 (33.7) |  |
| Cardiovascular | 13 (48.1) | 42 (44.2) |  |
| Orthopedics | 1 (3.7) | 2 (2.1) |  |
| Urology | 3 (11.1) | 5 (5.3) |  |
| Body weight, kg, median [IQR] | 63.20 [55.00, 73.00] | 62.00 [53.80, 69.15] | 0.38 |
| Height, cm, mean (SD) | 164.26 (10.44) | 161.66 (8.43) | 0.18 |
| Body mass index, kg/m^2^, median [IQR] | 23.88 [21.13, 26.08] | 23.43 [21.09, 26.16] | 0.69 |
| Duration of surgery, minutes,  median [IQR] | 265.00 [171.00, 341.00] | 264.00 [166.00, 369.50] | 0.77 |
| APACHE2 score (median [IQR]) | 12.00 [9.00, 12.00] | 12.00 [10.00, 14.00] | 0.42 |
| Antidiabetic Medications |  |  |  |
| Type of SGLT2 inhibitor, n (%) |  |  |  |
| Ipragliflozin | 2 (7.4) | 0 (0.0) |  |
| Empagliflozin | 21 (77.8) | 0 (0.0) |  |
| Canagliflozin | 2 (7.4) | 0 (0.0) |  |
| Dapagliflozin | 1 (3.7) | 0 (0.0) |  |
| Luceogliflozin | 1 (3.7) | 0 (0.0) |  |
| Insulin, n (%) | 16 (59.3) | 42 (44.2) | 0.19 |
| Metformin, n (%) | 6 (22.2) | 15 (15.8) | 0.56 |
| Sulfonylurea, n (%) | 7 (25.9) | 22 (23.2) | 0.8 |
| Dipeptidyl peptidase-4 inhibitor, n (%) | 17 (63.0) | 62 (65.3) | 0.82 |
| Thiazolidine, n (%) | 1 (3.7) | 2 (2.1) | 0.53 |
| GLP1 receptor agonist, n (%) | 2 (7.4) | 7 (7.4) | 1 |
| Alpha-glucosidase inhibitor, n (%) | 4 (14.8) | 8 (8.4) | 0.46 |
| Glinide, n (%) | 3 (11.1) | 4 (4.2) | 0.18 |
| Previous-onset comorbidities |  |  |  |
| Cancer, n (%) | 13 (48.1) | 54 (56.8) | 0.51 |
| Hypertension, n (%) | 18 (66.7) | 45 (47.4) | 0.09 |
| Heart failure, n (%) | 9 (33.3) | 13 (13.7) | **0.03** |
| Myocardial infarction, n (%) | 16 (59.3) | 39 (41.1) | 0.13 |
| Stroke, n (%) | 4 (14.8) | 8 (8.4) | 0.46 |
| Peripheral arterial disease, n (%) | 5 (18.5) | 10 (10.5) | 0.32 |
| Other kidney disease, n (%) | 0 (0.0) | 4 (4.2) | 0.58 |
| Maintenance dialysis, n (%) | 0 (0.0) | 0 (0.0) | NA |

Abbreviation: HbA1c = hemoglobin A1c, eGFR = estimated glomerular filtration rate, APACHE2 = Acute Physiology and Chronic Health Evaluation II, SGLT2 = Sodium-Glucose Cotransporter-2, GLP1 = glucagon-like peptide-1, IQR = interquartile range, SD = standard deviation

Significant *p*-values (*p* < 0.05) are given in bold.
