# Supplementary material for: The effect of preoperative sodium-glucose cotransporter 2 inhibitors on the incidence of perioperative metabolic acidosis: A retrospective cohort study
Source: BMC Endocr Disord. 2022 Aug 20;22:209. doi: 10.1186/s12902-022-01126-z (PMC9392326; doi:10.1186/s12902-022-01126-z)
Supplement: Supplementary file 3 — Additional file 3: Supplemental Digital Content 3. Multivariable linear regression analysis to identify factors associated with the lowest pH during intensive care unit stay. [file 12902_2022_1126_MOESM3_ESM.docx]

Supplemental Digital Content 3. Multivariable linear regression analysis to identify factors associated with the lowest pH during intensive care unit stay

| Variable | Coefficient | *p* value | 95% Confidence Interval | Variance inflation factor |
| --- | --- | --- | --- | --- |
| (Intercept) | 7.64 | <0.01 | 7.55 to 7.73 | Not applicable |
| Use of insulin | -0.0063 | 0.32 | -0.019 to 0.006 | 1.32 |
| Use of SGLT2 inhibitor | -0.026 | **< 0.01** | -0.041 to -0.01 | 1.25 |
| Use of metformin | 0.013 | 0.12 | -0.004 to 0.029 | 1.15 |
| eGFR | -0.00001 | 0.94 | -0.0003 to 0.0003 | 2.09 |
| Age | 0.0008 | 0.08 | -0.00009 to 0.0016 | 1.52 |
| Duration of surgery | -0.0000485 | **0.04** | -0.00009 to -0.000002 | 1.56 |
| PaCO_2_ | -0.006 | **<0.01** | -0.007 to -0.005 | 1.29 |
| Anion gap | -0.006 | **< 0.01** | -0.0079 to -0.004 | 1.54 |
| Hemoglobin A1c | 0.0046 | 0.07 | -0.0004 to 0.0095 | 1.34 |
| APACHE II | -0.0035 | **<0.01** | -0.0052 to -0.001 | 1.69 |
| Dialysis | 0.012 | 0.35 | -0.012 to 0.04 | 2.30 |
| Surgical site: thoracic | -0.0041 | 0.71 | -0.026 to 0.018 | 3.09 |
| Surgical site: cardiovascular | -0.0034 | 0.71 | -0.021 to 0.014 | 2.69 |
| Surgical site: orthopedics | 0.0075 | 0.71 | -0.032 to 0.047 | 1.37 |
| Surgical site: urology | 0.0056 | 0.67 | -0.020 to 0.030 | 1.69 |

Abbreviation: SGLT2 = Sodium-Glucose Cotransporter-2, eGFR = estimated glomerular filtration rate, PaCO_2_ = partial pressure of arterial carbon dioxide, APACHE2 = Acute Physiology and Chronic Health Evaluation II

The surgical site was analyzed as a categorical variable, and the analysis was conducted using “Surgical site: abdomen” as the reference.

Significant values (p < 0.05) are given in bold.
